# N, N′-Olefin Functionalized Bis-Imidazolium Gold(I) Salt Is an Efficient Candidate to Control Keratitis-Associated Eye Infection

**DOI:** 10.1371/journal.pone.0058346

**Published:** 2013-03-15

**Authors:** Tapastaru Samanta, Gourisankar Roymahapatra, William F. Porto, Saikat Seth, Sudipta Ghorai, Suman Saha, Jayangshu Sengupta, Octávio L. Franco, Joydev Dinda, Santi M. Mandal

**Affiliations:** 1 School of Applied Sciences, Haldia Institute of Technology, Haldia, West Bengal, India; 2 Central Research Facility, Indian Institute of Technology Kharagpur, Kharagpur, West Bengal, India; 3 Centro de Análises Proteômicas e Bioquímicas, Pós-Graduação em Ciências Genômicas e Biotecnologia UCB, Brasília-DF, Brazil; 4 Mugberia Gangadhar College, Egra Sarada Sashibhusan Mahavidyalaya, West Bengal, India; 5 Priyamvada Birla Aravind Eye Hospital, Kolkata, West Bengal, India; MGH, MMS, United States of America

## Abstract

Keratitis treatment has become more complicated due to the emergence of bacterial or fungal pathogens with enhanced antibiotic resistance. The pharmaceutical applications of N-heterocyclic carbene complexes have received remarkable attention due to their antimicrobial properties. In this paper, the new precursor, 3,3′-(p-phenylenedimethylene) bis{1-(2- methyl-allyl)imidazolium} bromide (**1a**) and its analogous PF_6_ salt (**1b**) were synthesized. Furthermore, silver(I) and gold(I) -N-heterocyclic carbene (NHC) complexes [Ag_2_LBr_2_/Au_2_LBr_2_; **2a**/**3a**], [(Ag_2_L_2_)(PF_6_)_2_/(Au_2_L_2_)(PF_6_)_2_; **2b**/**3b**] were developed from their corresponding ligands. All compounds were screened for their antimicrobial activities against multiple keratitis-associated human eye pathogens, including bacteria and fungi. Complexes **2a** and **3a** showed highest activity, and the effectiveness of **3a** was also studied, focusing eradication of pathogen biofilm. Furthermore, the structures of **1a**, **2a** and **3b** were determined using single crystal X-ray analysis, **2b** and **3a** were optimized theoretically. The mechanism of action of **3a** was evaluated by scanning electron microscopy and docking experiments, suggesting that its target is the cell membrane. In summary, **3a** may be helpful in developing antimicrobial therapies in patients suffering from keratitis-associated eye infections caused by multidrug-resistant pathogens.

## Introduction

Keratitis is a common corneal infection in tropical areas of the world. This kind of infection is quite dangerous, and in many cases can lead to permanent blindness, specially if not diagnosed promptly and treated effectively. Keratitis can develope from fungal (*Candida, Aspergillus, Fusarium* etc.) and bacterial (*Staphylococcus aureus, Pseudomonas aeruginosa* etc.) infections, especially those caused by the use of contact lenses or by eye injuries [1], [2]. The emergence of bacterial and fungal pathogens with enhanced antibiotic resistance has arisen due to a number of reasons, such as mutations, gene transfer, biofilm formation and inappropriate use of traditional antibiotics [3], [4]. The association of antibiotic-resistant pathogens with keratitis infection is a matter of great concern, since many infections have had no effective treatment yet.

Since antibiotic resistance seems to be inevitable, strenuous efforts have been made to develop new antimicrobial agents. The pharmaceutical application of N-heterocyclic carbene (NHC) and their metal complexes have gained enormous attention due to their antimicrobial properties. Recently, pyrazine functionalized-NHC complexes with clear deleterious effects against multidrug-resistant pathogens were seen to show an unusual mechanism of action [5]. Among them, gold (I) and silver (I)-NHCs seem to be remarkable candidates for antibiotic development, due to their higher activity and relatively low toxicity to mammalian cells when compared to other metals, such as Ru(II), Ru(I), Cu(I) and Pd(II)-NHC complexes. Hindi *et al.* [6] summarized the antimicrobial activities of several NHCs showing clear benefits, but demonstrated the need for higher amounts in order to obtain effective pathogen control. However, NHCs are in a versatile ligand class which has the advantage of easy manipulation. Furthermore, they also have the ability to bind simply to metals and can be readily functionalized [7]. Ag(I) and Au(I)−NHC complexes have also been found in many medicinal applications including antimicrobials [8]. Gold *in vivo* biochemistry still remains enigmatic, mainly due to a scarcity of adequate models and an incomplete understanding of gold reactivity. Imidazolium salts react with silver oxide and produce an NHC silver complex, which serves as a useful trans-metallating reagent [9] which has been widely studied. Finally, an interesting aspect of silver (I)-NHC complexes is the aggregation of silver (I) centers by argentophilic attraction with Ag–Ag separations [10] shorter than the sum of their van der Waals radii.

Research in this area is made easier and more interesting by the fact that steric and electronic properties as well the reactivity of metal–NHC complexes can be studied by varying the N-substituents of imidazole [11]. Many NHC complexes have been reported to include NHCs with simple N-alkyl or aryl substituents. Various functional groups [5] such as pyridyl, phosphinyl, pyrazolyl and thiazole have also attracted considerable attention due to their use in constructing N-functionalized NHCs. In this paper, we look at this reaction, and report the synthesis of a new type of silver-NHC complex. The hemi-labile nature of the allylic [12] system changes the metal-organic coordination mode during the formation of the complex, which encouraged us to design Ag and Au-complexes of the allylic ligand.

Data reported here show the antimicrobial activity of N, N′-olefin functionalized bis-imidazolium gold (I) and silver (I) salts against several resistant human fungal and bacterial pathogens, which can cause the serious eye infection keratitis. Their synthesis, crystal structures, characterization, and mode of action were analyzed and *ex vivo* bioassays were carried out.

## Materials and Methods

### Reagents and Chemicals

All the reagents - imidazole, α,ά - dibromo p-xylene, 3-chloro-2-methyl propene, Ag_2_O and NH_4_PF_6_ - were purchased from Sigma-Aldrich, UK, and were used without further purification. All manipulations were carried out in an open atmosphere unless otherwise stated. Au(SMe_2_)Cl was prepared according to the reported procedure [5]. Solvents EtOH, MeOH and CHCl_3_ of analytical-grade type were distilled and dried over appropriate drying agents and dinitrogen prior to use. All the selected antibiotics and antifungals tested in this study were purchased from HIMEDIA, India. ^1^HNMR spectra were recorded on a Bruker ARX 400 in (CD_3_)_2_SO_2_ as the solvent and^ 13^CNMR data were recorded on 75 MHz instrument respectively and referenced to residual protons and ^13^C signals of deuterium solvents. MALDI MS spectra were recorded on a Applied Biosystem, Voyager time-of-flight mass spectrometer (Text **S4**).

### Synthesis of Compounds

A solution of (680 mg, 10 mmol) imidazole and (400 mg, 10 mmol) NaOH was prepared in THF, 3-Chloro-2-methyl propene (905 mg, 10 mmol) was added to the solution and the mixture was refluxed for 15 h at 65°C. The reaction mixture was cooled at room temperature and the solvent was removed. After removal of the solvent, a brown, oily liquid was isolated and characterized as 1-(2-methyl allyl) imidazole. Then 1-(2-methyl-allyl)imidazole (244 mg, 2 mmol) and α,ά - dibromo p-xylene (264 mg, 1 mmol) was added to the reaction mixture (neat reaction), heated for 6 h producing a colorless solid. The solid mass was washed with Pet-ether and recrystallized in MeOH. 3,3′-(p-phenylenedimethylene) bis{1-(2- methyl-allyl)imidazolium} hexafluorophosphate salt (**1b**) was synthesized using the aqueous solution of ammonium hexafluorphosphate (NH_4_PF_6_) in complex (**1a)** and produces the colorless, water-insoluble hexafluorphosphate salt (**1b**).

Furthermore, 3,3′-(p-phenylenedimethylene) bis{1-(2- methyl-allyl)imidazoline}silver bromide (**2a**) salt was synthesized after a reaction of complex **1a** (102 mg, 0.2 mmol) and Ag_2_O (47 mg, 0.2 mmol) in dichloromethane, and the mixture was stirred at room temperature for 12 h with exclusion of light, then filtered to remove a small amount of unreacted Ag_2_O. The filtrate was collected and solvent was removed producing a white solid mass (**2a**). Furthermore, 144 mg (0.2 mmole) of solid silver bromide complex (**2a**) was taken in a round-bottomed flask and dissolved in 20 mL dichloromethane solvent. Au(SMe_2_)Cl (59 mg, 0.2 mmol) was added to it in N_2_ medium and stirred for 1 h, and precipitation of AgCl was observed. It was then filtered and the filtrate was collected; after removal of the solvent, the colorless solid gold complex produced 3,3′-(p-phenylenedimethylene) bis{1-(2- methyl-allyl)imidazoline}gold(I) bromide (**3a**) salt.

3,3′-(p-phenylenedimethylene) bis{1-(2- methyl-allyl)imidazoline}silver hexafluorophosphate (**2b**) was synthesized by the reaction of complex (**1b)** (64 mg, 0.1 mmol) with excess Ag_2_O (27 mg) in acetonitrile (CH_3_CN). The mixture was stirred at room temperature for 8 h with exclusion of light and then filtered to remove a small amount of unreacted Ag_2_O. The filtrate was collected and solvent was removed to produce a white solid mass of complex (**2b)**. 90 mg (0.15 mmole) of solid silver hexafluorophosphate complex (**2b**) was put into a round-bottomed flask and dissolved in 10 mL of acetonitrile solvent. Au(SMe2)Cl (45 mg, 0.15 mmol) was added to (**2b**) in N_2_ medium and stirred for 1 h. Precipitation of AgCl was observed, which was then filtered. Filtrate was collected and, after removal of solvent, produced the colorless solid 3,3′-(p-phenylenedimethylene) bis{1-(2- methyl-allyl)imidazoline}gold(I) hexafluorophosphate complex (**3b**). The chemical shift (^1^HNMR and ^13^CNMR) and mass (m/z) data of all synthesized compounds were described in the supplementary file.

### X-Ray Structure Determinations

Crystals of **1a, 2a** and **3b** were coated in paraffin oil, mounted on a kryo loop, and placed on a goniometer at a temperature of 296 K in an open atmosphere. X-ray data sets were collected using a Bruker Apex CCD diffractometer with graphite monochromated MoK \ α radiation (λ = 0.71073 Å ). The unit cell determination was achieved by using reflections from three different orientations. An empirical absorption and crystal refinement corrections were performed by using multiscan SADABS. Structure solution, refinement and modeling were accomplished by using the SHELXL-97 package [13]. The structure was obtained by full matrix least square refinement of F^2^ and selection of appropriate atoms from the generated difference map. Additional data collection and refinement parameters are summarized in **Table S1** and **S2**.

### Structure Optimization

In order to understand the structure of synthesized complexes **2b** and **3a**, the structures were drawn according to the NMR and mass data found, and also further generated the structures of complexes **2a** and **3b.** Geometries at the B3LYP/LANL2DZ level of theory using the Gaussian G-03-E01 [14] program were optimized. The number of imaginary frequencies of all the molecules turns out to be zero, implying that they correspond to minimum energy structures on the potential energy surface.

### Microbial Strains and Growth Conditions

The *Aspergillus fumigatus* JM3 (filamentous fungus), *Candida albicans* SJ11 (unicellular fungus) *Pseudomonas aeruginosa* (Gram-negative bacteria), and *Staphylococcus aureus* (Gram-positive bacteria) used in this study were obtained from Priyamvada Birla Aravind Eye Hospital in Kolkata, India. The phenotypic characteristics of clinically isolated fungal strains and antibiotic sensitivity profiling of bacterial strains were described in the supplementary section. All the strains were isolated from patients associated with keratitis, and fungal strains were previously characterized [2], [15]. Fungal species were collected from a mature solid medium culture plate (Sabouraud dextrose agar) and mixed with liquid RPMI 1640 (Himedia, India), and were incubated for 24 h at 30°C to get the relevant turbidity of 0.5 ×10^4^ CFU/mL. Bacterial cultures were maintained in Mueller-Hinton Broth (MHB) and a total inoculum load of ca. 10^5^ cells per well-maintained.

### Antimicrobial Assays

Minimum inhibitory concentration (MIC) values of all the synthesized compounds and antifungal/antibacterial antibiotics were determined according to CLSI guidelines [16]. The concentration of each compound used for the assay ranged from 0.97 µM to 1 mM. MIC values were determined where no visible growth was observed. The characteristic features of clinical isolates with their antimicrobial sensitivity were described in Text **S3**. The culture conditions and bacterial growth were monitored as described earlier by Roymahapatra *et al.* [5]. Likewise, fungal growth and culture conditions were monitored as described earlier, following Sengupta *et al.* [2]. Two wells, where no compounds were added, were used as positive controls, and compounds with the absence of microorganisms were used as negative controls in order to maintain the experimental sterility, and all independent experiments were repeated four times.

To examine the bacterial growth or killing kinetics in the presence of compound **3a**, bacterial cells were grown in 100 mL of Mueller-Hinton Broth (MHB) supplemented with different doses of complex (0.25 to 4.0 µM), at 37°C with continuous agitation at 180 rpm. Growth or killing rates and bacterial concentrations were determined by measuring OD at 600 nm. The OD values were converted into concentration of cells measured in CFU per milliliter (1.0 OD corresponded to 2.16×10^8^ CFU/mL). *In vitro* growth kinetics of fungal strains were determined following the protocol described by Mitchell *et al.* [17]. In brief, fungal strains (10^3^ CFU) were inoculated into 2 mL Sabouraud dextrose broth supplemented with different doses of complex **3a** (0.25 to 4.0 µM), and incubated at 30°C with continuous rocking at 180 rpm. Two milliliters of mock-inoculated Sabouraud dextrose broth served as a negative control. The *in vitro* growth rates were measured at OD_600_ and using the conversion factor of 3×10^7^ CFU/mL per 1 U OD_600_ [17]. All independent experiments were repeated four times.

### Scanning Electron Microscopy (SEM)

*Pseudomonas aeruginosa* and *Candida albicans* cells were harvested from the log phase of their respective growth medium. The cells were then washed three times with 1X PBS buffer and resuspended in the same saline buffer. The cells were treated with gold (I) complex (**3a**) with their individual MIC concentration for 30 min. Cells were repeatedly washed with saline water and 5–10 µl of resuspended solution containing bacteria/fungus was placed on the lysine-coated glass cover slip as a drop-caste method. The fixed cells were dried and kept on desiccators until use. Samples were then fixed onto a graphite stub and kept in an auto sputter coater (E5200, Bio-Rad) under low vacuum for gold coating up to 120 seconds. Surface morphology was studied by using a scanning electron microscope (JEOL JSM5800) with an accelerated voltage between 5–20 kV.

### Eradication of Biofilm on Contact Lenses

The formation of *Aspergillus fumigatus* and *Candida albicans* biofilm on contact lenses was carried out according to Sengupta *et al.* [2]. Soft contact lenses were separately submerged in 6 wells of flat-bottomed polystyrene plates containing 2 mL of RPMI 1640 medium, inoculated with the respective fungal strain with an inoculum dose of 3.5×10^6^ CFU/mL and incubated for 72 h at 30°C. After 72 h, the lenses were removed and the planktonic cells were washed gently and repeatedly with 1X PBS buffer. To determine the effect of gold (I) complex (**3a**) on biofilm eradication of the contact lenses, complexes were added to the biofilm in serial double-diluted concentrations (0.97 µM to 1 mM), and the final volume of 2 mL with RPMI medium, which was then incubated further for 24 h at 30°C. A series of complex-free wells and biofilm-free wells was also included to serve as positive and negative controls, respectively. After incubation, the remaining biofilm metabolic activity was quantified by the XTT-reduction assay as described earlier [13]. For imaging, the lenses were flipped, and stained biofilms were visualized by fluorescence microscopy to compare their gross morphologies. Live-dead analysis was performed with BacLight Live/Dead dye (Invitrogen Inc. USA). The images were captured from the stained biofilms under fluorescence microscope (OLYMPUS IX 51, fitted with Evolution VF CCD Camera).

Biofilm formation by bacterial clinical isolates of *P. aeruginosa* and *S. aureus* on contact lenses were determined by using a polystyrene crystal violet adherence assay, as described previously by Croes *et al.* [18]. In brief, soft contact lenses were separately submerged in 6 wells of flat-bottomed polystyrene plates containing 2 mL of Trypticase Soy Broth (TSB) with 0.5% glucose, inoculated with the respective bacterial strain with an inoculum dose of 10^8^ CFU/mL and incubated for 48 h at 37°C. The biofilm that had grown over contact lenses was used for minimum biofilm eradication concentrations (MBEC) assay following Roymahapatra *et al*. [5] and complex concentrations were used in the same way as in the previous fungal assay. The crystal violet retained within the biofilm was extracted by adding 100** µ**l of 70% (vol/vol) ethanol with 10% isopropyl alcohol (vol/vol) and measured at 590 nm (**A**590). All independent assays were performed four times. Subsequently, the biofilm formations in different concentrations were visualized under fluorescence microscope (OLYMPUS IX 51, fitted with Evolution VF CCD Camera) by staining with BacLight Live/Dead dye (Invitrogen Inc. USA).

### Cytoplasmic Material Release Study

Cytoplasmic material release study was carried out according to Sahu *et al*. [19], with minor modifications. To summarize, 1 mL of overnight bacterial (*P. aeruginosa* and *S. aureus*) and fungal (*C. albicans*) culture (relevant turbidity of 0.5 ×10^4^ CFU/mL) were centrifuged and the pellet was washed with PBS (1X) buffer. Next, the pellet was suspended in 1 mL of PBS (1X) buffer and divided into five aliquots of 200 µL each. Different aliquots of cell suspensions were treated with different concentration of complex 3a (0.5 to 4 µM) and incubated at 30°C for 2 h. The samples were filtered to remove bacteria, and OD values of supernatants were recorded at 260 nm. The percentage of cytoplasmic material release was measured considering the OD values obtained with TrintonX-100 (0.1%) treatment with 100% release. The experiment was repeated three times.

### Molecular Docking

Two membrane models were built through the CHARMM-GUI server [20] for simulating fungal and bacterial membranes, both with 50 Å^2^. For fungal membrane, a proportion of 1∶1∶1∶1∶1∶1 of POPA (palmitoyloleoylphosphatidylamine), POPS (palmitoyloleoylphosphatidylserine), POPE (palmitoyloleoylphosphatidylethanolamine), DOPC (dioleoylphosphatidylcholine), DPPC (dipalmitoylphosphatidylcholine) and cholesterol was used according to Jo *et al*. [20]. For the bacterial membrane, a 3∶1 proportion of of POPE and POPG (phosphatidylglycerol) was used according to Murzyn *et al*. [21].

Molecular docking studies were performed by AutoDock Vina and AutoDock Tools as previously described [5], [22]–[24]. Grid boxes were set to the center of the membrane models and then dislocated to cover one layer of membrane models, while for other receptors the grid boxes were kept in the center of the receptor. The grid box sizes for membrane models were set to 20, 20 and 30 for X, Y and Z axes, respectively. The **3a** structure was set as a flexible ligand by using the default parameters of the AutoDock Tools. Final models were analyzed on PyMol (The PyMOL Molecular Graphics System, Version 1.4.1, Schrödinger, LLC).

### MTT Assay

Non-carcinoma mouse embryo fibroblast cell (3T3) and human breast carcinoma cell (MDA-MB-231) lines were used in this study. The cells were cultured as monolayers in Dulbecco’s Modified Eagle Medium (DMEM) supplemented with 10% (v/v) heat-inactivated fetal bovine serum and antibiotics. After overnight incubation, the medium was aspirated off, washed with PBS (1X) buffer and 200 µL of complex **2a**, and **3a** (0–100 µM concentration) medium were added separately in triplicates. The MTT [3-(4,5-dimethylthiazol-2-yl)-2,5-diphenyltetrazolium bromide] assay was performed following the method described by Mandal *et al*. [25].

### Compatibility Test

Ophthalmologists generally have a large repertoire of antibacterial and antifungal antibiotics in the form of eye drops, ointments, tablets and parenteral applications to control eye infections. Complex **3a** was used in an eye drop and tested for 48 h, six times a day on goat (*Capra aegagrus hircus*) eyes, and the concentration was 5-fold of that used in *in vitro* experiments. The goat eyes were collected from a butcher’s shop at the local market. In general, enucleation of the entire globe of the eyes was done after death. The cornea was excised from globe under an aseptic condition in a biosafety cabinet. Next, the excised cornea was kept in a McCarey-Kaufman medium for preservation without any antibiotics. Experiments were repeated with three eyes. Each eye was kept at 4°C in a petri dish and drops were applied at 4 hour intervals. After 48 hours of treatment, the blubber conjunctiva, retina and lens were evaluated for any damage, cracking or dilution by visual observation.

### Statistical Analysis

The results are presented as the mean ± SD. The statistical significance of the experimental results was determined by one-way Student’s *t*-test or one-way ANOVA followed by Dunnett’s test. Values of p<0.05 were considered statistically significant. Prism version 5.0 was used for all statistical analyses.

## Results

### Synthesis of the Complexes

**Figure 1:**
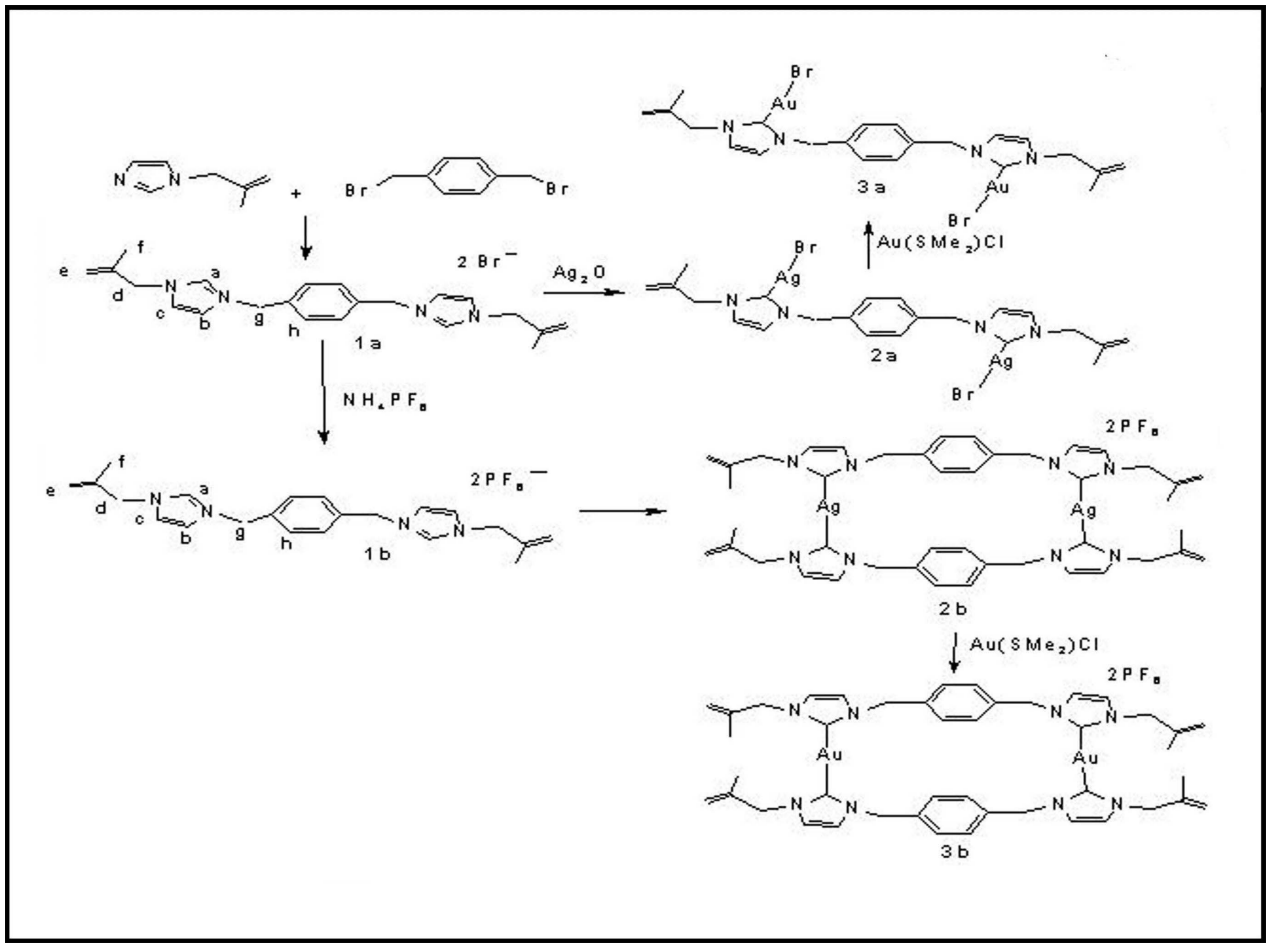
Schematic diagram of the synthesis procedure of ligands 1a and 1b, and their derived compounds 2a, 3a and 2b, 3b.

The ligand **1a** was synthesized by the addition of imidazole and NaOH in dry THF under stirring conditions at room temperature and a subsequent addition of 3-chloro-2-methylpropene. The mixture was refluxed at 65°C for 15 h and allowed to cool at room temperature, and the solvent was removed. A brown oily liquid form of 1-(2-methyl allyl)imidazole was obtained. Then 1-(2-methyl-allyl)imidazole and α,ά – dibromo- p-xylene were mixed and the reaction mixture was heated for 6 hours to obtain a colorless solid **1a** (Yield: 490 mg, 0.96 mmol, 96%). Quantitative conversion of PF_6_ salt (**1b**) of the proligand was achieved by the addition of aqueous solution of ammonium hexafluorphosphate in solution **1a.** The presence of NCHN proton in ^1^H NMR spectra confirmed the formation of ligands **1a** (δ = 9.47 ppm) and **1b (**δ = 9.25 ppm). Elemental analysis, ^13^CNMR and MALDI mass spectra support the formulation of proligands **1a** and **1b**. Complex **2a** was prepared (Yield of **2a**: 115 mg, 0.15 mmol, 81%) by the complexation of proligand- **1a** and Ag_2_O in dichloromethane with exclusion of light (**Fig.** **1**) as reported in the general procedure [5]. Complex **3a** was prepared by the silver carbene transmetallation [5] method (Yield of **3a**: 135 mg, 0.17 mmole, 83%). Afterward, complex **2a** and Au(SMe_2_)Cl were combined in DCM under nitrogen atmosphere at room temperature. Complex **2b** was prepared by mixing proligand **1b** with Ag_2_O in acetonitrile under stirring (4 h) at room temperature (Yield of **2b**: 52 mg, 0.085 mmol, 86%). **3b** was also prepared by a transmetallation method using **2b** and Au(SMe_2_)Cl in acetonitrile (Yield of **3b**: 94 mg, 0.14 mmol, 92%). Formation of the complexes **2a**, **2b**, **3a** and **3b** was confirmed by the absence of imidazolium protons (C_2_H) and a downfield shift of aromatic protons. In the case of **2a,** the carbenic signal appeared at 178.2 ppm in the ^13^CNMR spectra, and a downfield shift of 38.9 ppm in comparison to free imidazolium salt was observed after complexation. A significant 37–39 ppm shift of carbenic carbon in the ^13^CNMR spectrum confirmed the formation of complexes **2b, 3a** and **3b**. This was further supported by the mass spectrometric data. The single crystal X-ray structures of **1a**, **2a** and **3b** were determined (see supplementary file). ^1^HNMR,^ 13^CNMR and MALDI mass spectra of all synthesized complexes were supplied in the supplementary file and their corresponding structures were determined (Fig. **S1** **to** **S5,** Text **S1** and **S2**).

### Antimicrobial Activity

**Figure 2:**
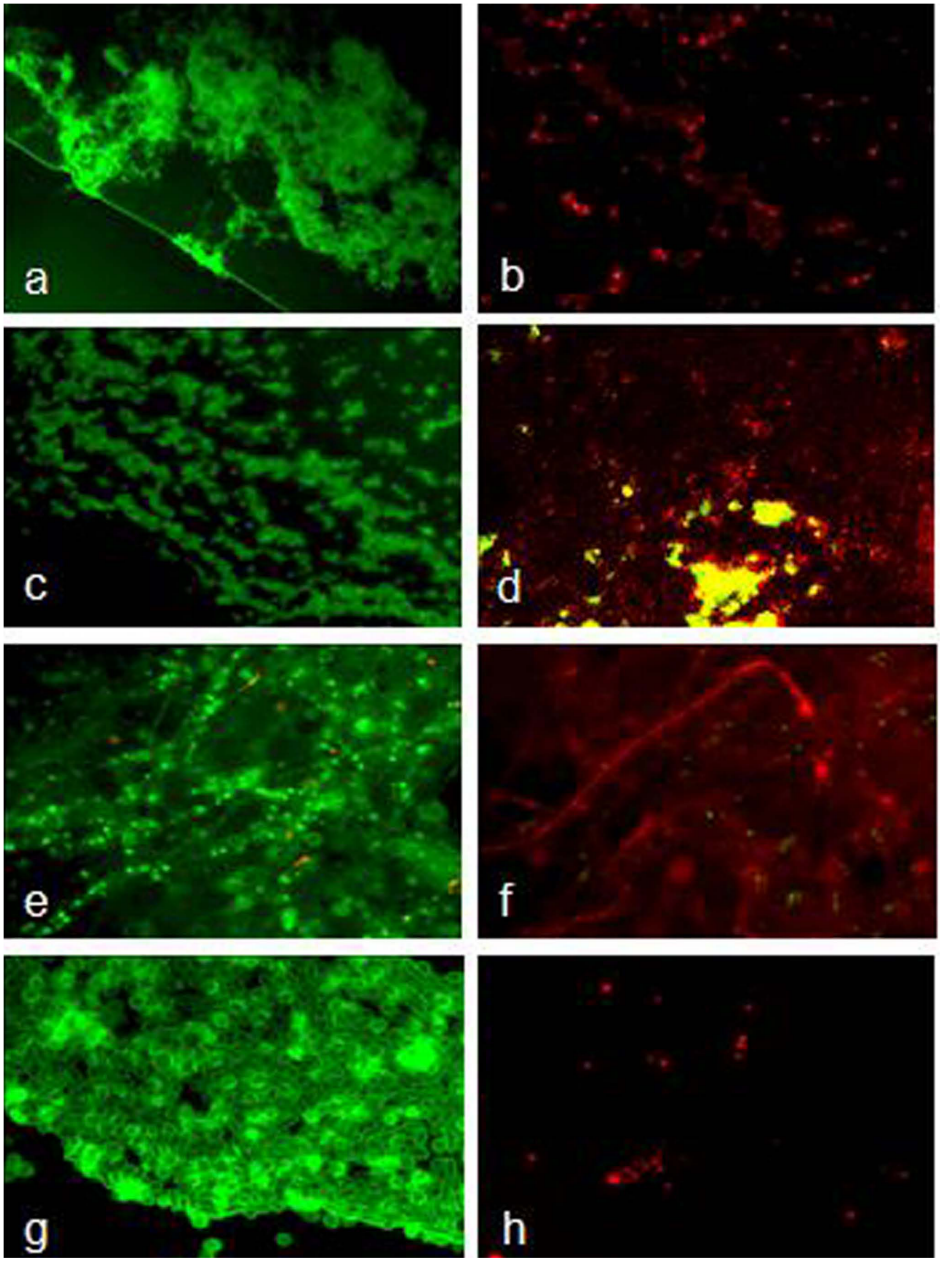
Images of biofilm eradication from soft contact lenses by complex 3a. Images were captured after 24 h treatment with 0.1% DMSO (left panel) and with complex **3a** (as MBEC values listed in **Table 2**) over 72 h-grown biofilm (right panel). Bacterial pathogens were *P. aeruginosa* (a & b); *S. aureus* (c & d) and fungal pathogens were *A. fumigetus* (e & f); *C. albicans* (g & h). Complex **3a** significantly reduced live cells (green fluorescence) and most of the cells were dead (red fluorescence) against all tested pathogens on contact lenses. Images were captured at a magnification of 400X.

The antibacterial and antifungal activities of all synthesized compounds were tested against both Gram-positive and -negative bacteria as well as against unicellular and filamentous fungus. The antibacterial activities of complexes **2a** and **3a** were higher than **2b** and **3b** (**Tables 1** **and** **2**). Complex **3a** was more active against *P. aeruginosa* and *A. fumigatus* than complex **2a** and other tested antibiotics. The eradication of pathogen-formed biofilm is an important criterion for the successful development of new generation antimicrobials. All the pathogenic strains were used to develop their biofilm over contact lenses and the biofilm eradication ability of complex **2a** and **3a** was checked. The minimum biofilm eradication concentrations of all the tested antibiotics, complex **2a** and **3a** were determined (**Table 2** **)**. The N,N′-olefin functionalized bis-imidazolium gold (I), **3a** salts are 3 to 10 fold more active than the silver (I) complex and commercial antibiotics. The fluorescence micrograph of control biofilm treated with complex **3a** revealed the effectiveness of biofilm eradication ability after 24 h of treatment (**Fig. 2**). The killing activity of complex **3a** for *P. aeruginosa* was different from *S. aureus* (**Fig. S6**). The killing activity was concentration-dependent and no cell growth was seen even after 0.5 and 2.5 µM concentration of complex **3a** for *P. aeruginosa* and *S. aureus,* respectively. Similarly, no cell growth was observed after 2.5 and 0.5 µM concentration of complex **3a** for *C. albicans* and *A. fumigatus,* respectively (**Fig. S7**).

**Table 1:** Minumum inhibitory concentration of the synthesized compounds, antibacterial and antifungal antibiotics against keratitis-associated eye pathogens.

Compounds	*C. albicans*	*A. fumigatus*	*P. aeruginosa*	*S. aureus*
2a	3.90	3.90	3.90	3.90
2b	125	125	125	125
3a	1.95	0.97	0.97	1.95
3b	125	125	125	125
Voriconazole	15.62	0.97	Nt	nt
Amphotericin B	1.95	0.97	Nt	nt
Fluconazole	31.25	500	Nt	nt
Ceftazidime	nt	nt	31.25	31.25
Vancomycin	nt	nt	7.81	15.62
Piperacillin	nt	nt	31.25	62.5

Data are representing as µM concentration. nt means “not tested”.

**Table 2:** Minimum biofilm eradication concentration of the synthesized compounds, antibacterial and antifungal antibiotics against keratitis-associated eye pathogens.

Compounds	*C.* *albicans*	*A.* *fumigatus*	*P.* *aeruginosa*	*S.* *aureus*
2a	15.62	7.81	7.81	7.81
2b	250	125	125	125
3a	3.90	1.95	1.95	3.90
3b	125	125	125	125
Voriconazole	125	125	nt	nt
Amphotericin B	31.25	15.62	nt	nt
Fluconazole	500	500	nt	nt
Ceftazidime	nt	nt	62.5	62.5
Vancomycin	nt	nt	31.25	31.25
Piperacillin	nt	nt	250	250

Data are representing as µM concentration. nt means “not tested”.

### Insights into Mechanism of Action

**Figure 3:**
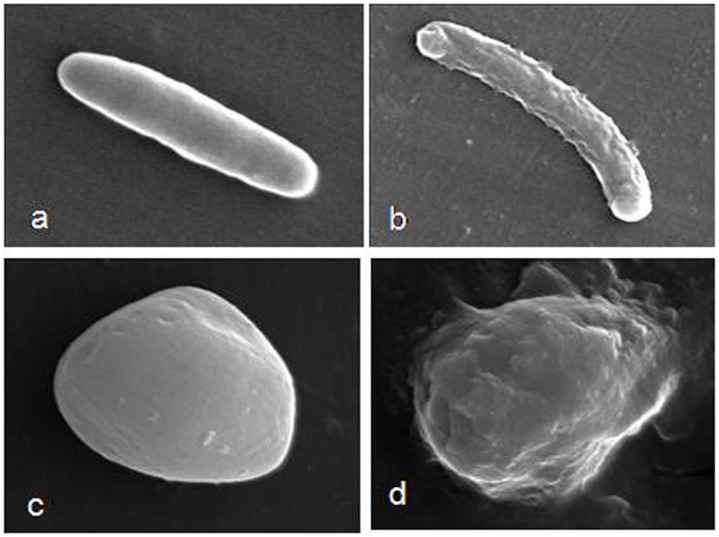
SEM micrographs of bacteria and fungi treated with compound 3a. Cells were treated with compound **3a** (2 µg.mL^−1^) and control group was treated with 0.1% DMSO. Control *P. aeruginosa* cells (a) and treated with **3a** (b); Control *C. albicans* cells (c) and treated with **3a** (d).

**Figure 4:**
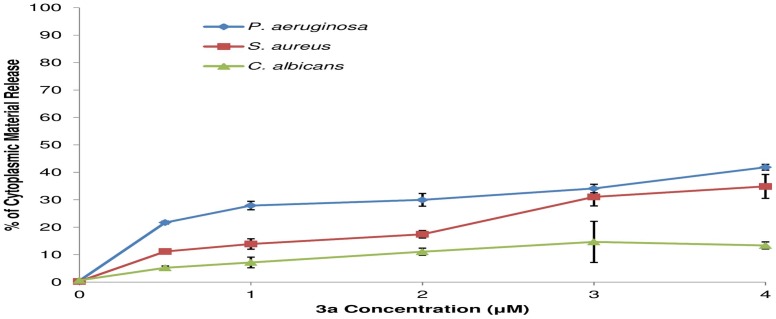
Effect of complex 3a on the release of cytoplasmic material at 260 nm. *P. aeruginosa* (blue line), *S. aureus* (red line) and *C. albicans* (green line) were incubated with different concentrations (0.5 to 5.0 µM) of complex **3a** at 30°C for 2 h. Release of cytoplasmic material was monitored at 260 nm wavelength and percent values were calculated in relation to triton treatment (positive control). The treatment with triton X-100 (0.1%) was considered as 100% of cytoplasmic material release. Data points represent mean and standard deviation of three experiments.

The interactions between microbial cell membrane and complex **3a** were observed by using a scanning electron microscope (SEM). SEM pictures clearly indicated a morphological change after 1 h of treatment with complex **3a** (**Fig. 3**). The morphological change indicates that the target should be found on the microorganism surface such as lipid bilayer or cell walls. In order to determine the morphological changes observed in SEM images were due to the membrane damage or not, the leakage of cytoplasmic materials was monitored at 260 nm wavelength. The leakage of cytoplasmic materials is considered a characteristic indication of damage to the cytoplasmic membrane [19]. Different concentrations of complex **3a** were incubated with bacterial and fungal cell suspensions for 2 h. Cytoplasmic material leakage was observed after increasing the complex concentration, and maximum leakage was found in *P. aeruginosa* in comparison to *S. aureus* and *C. albicans* (**Fig. 4**), which indicates that the main target might be lipid bilayer.

**Figure 5:**
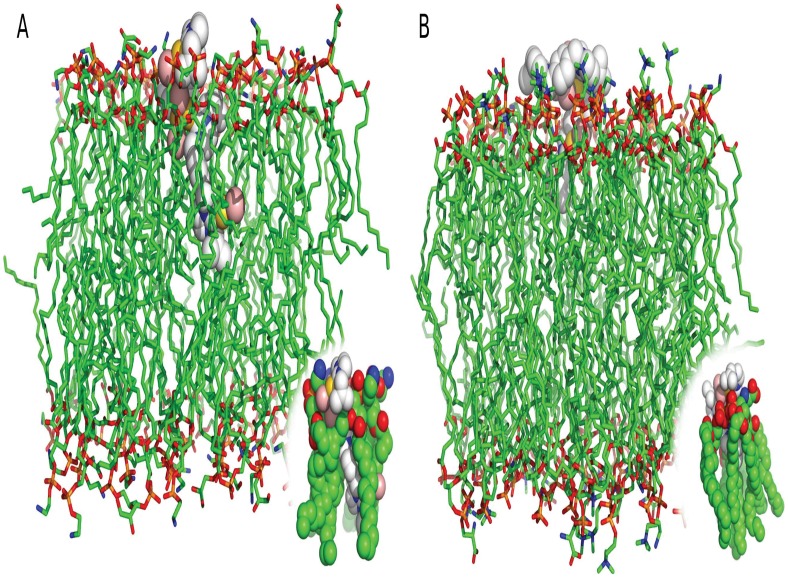
Theoretical docking studies of complex 3a with bacterium and fungus like membrane. Images are representing the docking results of complex **3a** with bacterium-like membrane (A) and fungus-like membrane (B). The membrane is represented in sticks, carbon atoms are in green, nitrogen atoms in blue, phosphorus atoms in orange and oxygen atoms in red. **3a** is represented in spheres: the gold atoms are in gold, carbon atoms in white, nitrogen atoms in blue and bromine atoms in pink. The interactions between the metal complexes and lipids are detailed at the bottom.

Therefore, docking experiments were performed in order to verify the binding affinities of **3a** compound to microbial cell membranes (**Fig.** **5**). Docking data shows the complexes formed between **3a** compounds with bacterium- and fungus-like membranes. The **3a** molecule is clearly more buried into bacterium-like membrane in comparison to fungus-like lipid bilayer. Moreover, docking results suggested that **3a** affinity to bacterium-like membranes is stronger than fungus-like membranes since the affinities were −7.4 kcal.mol^−1^ and −6.1 kcal.mol^−1^ respectively. Furthermore none polar interactions were observed in both cases, being complexation mainly driven by van der Walls forces.

### Compatibility Test

**Figure 6:**
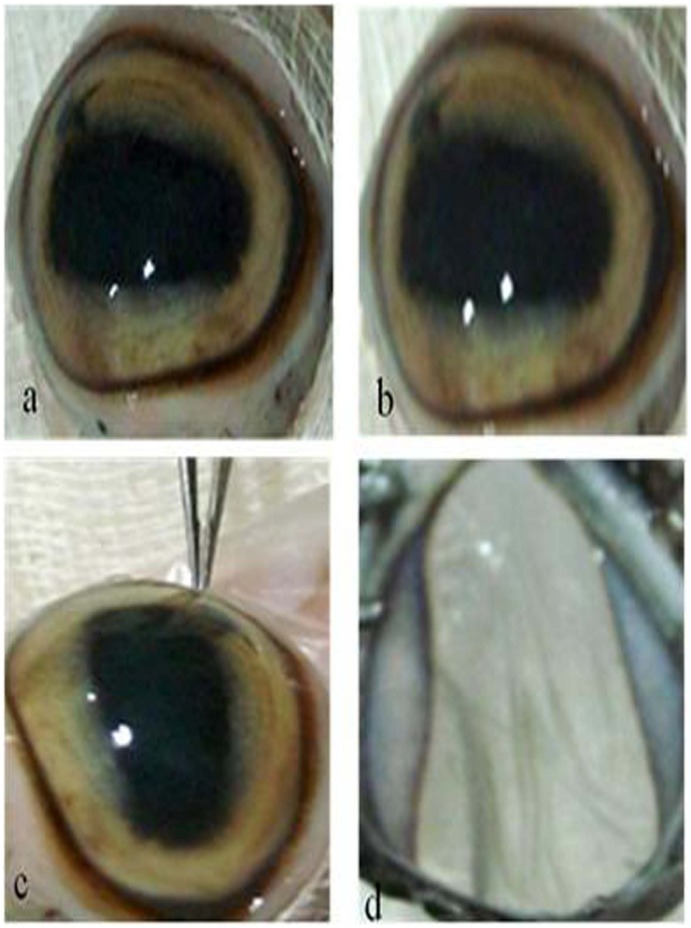
Compatibility test of complex 3a on goat (*Capra aegagrus hircus*) eyes, as an eye drop. Eye just excised from goat (a); after 48 h treated with compound **3a** (b), conjunctiva is not damaged or cracked after 48 h treatment with complex **3a** applied 6 times daily (c); and lens is neither diluted nor fractured after 48 h treatment (d). Images were captured at a magnification of 5X.

Both complexes **2a** and **3a** inhibited cell proliferation of MDA-MB-231 and 3T3 cells in a dose-dependent manner. Cell inhibition was prominent mainly at concentrations after 25 µM for both the complexes, as confirmed by MTT assay (**Fig. S8**). The IC_50_ values of complex **3a** against MDA-MB-231 and 3T3 cancer cells were 29.21±2.38 µM and 23.44±2.01 µM, and the values of **2a** against MDA-MB-231 and 3T3 cells were 32.94±2.72 µM and 26.21±1.88 µM, respectively. In addition, complex **3a** was used as an eye drop at five times the concentration observed in the *in vitro* experiment and applied onto the normal eye that had been excised from a goat. After 48 h of treatment with complex **3a**, which was applied 6 times daily, the complex was revealed to have low toxicity to the eye (**Fig. 6**). The pigment and lens were neither diluted nor fractured after 48 h of treatment with complex **3a**, which suggests its low toxicity and future application in keratitis control.

## Discussion

N,N′- olefin functionalized allylic ligands, 3,3′-(p-phenylenedimethylene) bis{1-(2- methyl-allyl)imidazolium}bromide(**1a**) and 3,3′-(p-phenylenedimethylene)bis{1-(2-methyl-allyl)imidazolium} hexafluorophosphate(**1b**) were used to synthesize four bis-imidazolium silver (I) and gold (I) salts as well as **2a, 2b** and **3a, 3b**. Syntheses of these compounds were confirmed by using NMR (^1^H and ^13^C) and MS analysis. Single-crystal X-ray diffraction data were collected for compounds **1a**, **2a** and **3b**. The molecules (**2a, 2b** and **3a, 3b)** possessed N-C_carbene_ bond distances N-C_carbene_-N angles in immidazole moity were comparable with other imidazolium systems. The carbene-Ag-carbene bond angle in **2a** was 170.83(15)° and C-Ag-Br bond angle was about 170.6–171.6 ° *i.e.* linear, whereas Ag-Ag-Br bond angle was nearly 90 °, which indicates the Ag-Ag bond was perpendicular to C-Ag-Br in a ‘T’ shape. It revealed that the complex was of the bis-carbene type with its olifinic system confirmed by its dinuclear crystallinity [Ag_2_(**1a**-H)_2_]^2+^ motif. The same structural geometry was found in the optimized structure of complex **3a**. For **2a,** Ag-Ag shortest bond distance was found to be 3.084(3)A° (Ag4-Ag1) [**Fig.** **S2**] and that of Au-Au for **3a** was 3.08415A°(Au110–Au112) [**Fig.** **S3**]. These metal-metal bond distances are shorter than the reported olefin systems and even shorter than the sum of their van der Waals radii [26]. The single crystal structure of complex **3b** was determined by XRD study and that of **2b** through theoretical optimization. **2b** and **3b** looked like a ‘chair’ with two ligands bonding by metal center from the opposite side.

The synthesized complexes **2a**, **2b**, **3a** and **3b** were evaluated for their antimicrobial properties against human pathogens causing keratitis infection. Among them, complexes **2a** and **3a** showed the strongest antimicrobial activities against resistant pathogenic strains. The bacterial strains were resistant to the commonly used antibiotics ceftazidime, vancomycin, and piperacillin. The fungal strains were also resistant to amphotericin B, fluconazole, and voriconazole. Several resistant mechanisms such as enzymatic inactivation, chemical modification, alteration of the antibiotic target site and efflux pumps are well reported [27]. The most promising feature of this research was that all resistant strains evaluated here were highly susceptible to complexes **2a** and **3a**. This leads us to suggest that complexes **2a** and **3a** follow different mechanisms in order to inhibit microorganism growth.

Inhibition of biofilm formation or eradication of grown biofilm is the most important feature of these complexes. Complex **3a** was able to eradicate the biofilm formed by bacterial and fungal strains both significantly and successfully. Biofilm is a complex matrix of exopolymeric substances of the organisms that prevent drugs from direct penetration. Hence, it is also believed that the ability of complex **3a** to penetrate the biofilm matrix at very low doses might be related to the presence of a dinuclear [Au2(**1a**-H)2]^2+^ olifinic motif. Indeed, gold complexes showed good and selective activity against both Gram-positive and -negative bacteria, as well toward fungi. It is apparent that the functionalization of the nitrogen atoms of the NHC ligands and the complexation with Au (I) at the C2 site might be the cause of higher antimicrobial activity. The difference in activities depends on several factors since these complexes are known to interact freely with cellular components when they are in a flexible co-planar [28] configuration and have rotational freedom [29]. The relative energy as a function of the torsional angle [5] is also a relative descriptor for the co-planarity and free rotation which endorse easy binding of the metal center of the complex with the active site. In the present study, among the four complexes, the metal centers of **2a** and **3a** are bonded with a single ligand (other side attached to Br atom), having scope for free rotation. This free rotation promotes a co-planar configuration within the reaction medium for the easy formation of complexes with cellular components. On the other hand, ligand dissociation is prohibited in the case of cationic tetrahedral Au (1) phosphine complexes as they gain stability through chelating. This is due to the high stability of the metal-phosphorus bond [30], metal-F bond and probably formation of H-bond with ‘F’ of PF6^−^, as is the case for **2b** and **3b.** Since metal centers are connected with two ligands, free rotation is prohibited and they are more stabilized through chelating with PF_6_^−^, so their reactivity is lower than **2a** and **3a.** Our results are also in agreement with an earlier report by Özdemir *et al*. [31] that imidazolium salt bearing Cl and Br salts is very active; but the same derivative completely lost its activity when the anion was changed to PF_6_ or BF_4_.

The probable mechanism of action consists in the bacterial and fungal membranes destabilization, reducing the structural integrity and leading to clear microorganism shape modification. The membrane’s destabilizing effect is probably given by insertions of multiple molecules of **3a**, intercalating the lipids and leading to membrane disruption (**Figs. 2** and **3**). However, **3a** was more active against bacteria than fungi. This difference could be related to two elements, the cell walls thickness and the membrane affinities. According to the docking affinities, the binding of **3a** is more favorable to bacterium-like membranes than fungus-like ones. For bacterium-like membranes, a binding affinity of −7.4 kcal.mol^−1^ was observed whereas the affinity for fungus-like ones was −6.1 kcal.mol^−1^. In addition, the **3a** activity is influenced by the composition of the cell wall, the compound must cross the cell wall before acting in the lipid bilayer. The differences between the amounts of leakage materials are due to the variations of their cell wall compositions. *P. aeruginosa,* is a Gram-negative bacterium with a thinner peptidoglycan layer which makes cytoplasmic leakage more likely compared to the thicker peptidoglycan layer of Gram-positive bacterium, *S. aureus*. Furthermore, in *C. albicans*, the main cell wall composition is chitin, which makes a rigid layer surrounding the membrane. The cytoplasmic material leakage is more prominent and more rapid in *P. aeruginosa* followed by *S. aureus*. The leakage was nominal in *C. albicans* (**Fig. 4**). In addition, a small mammalian cell toxicity degree was observed in their corresponding MIC values between 0.5 to 5.0 µM through the MTT assay.

However, a question arises: why was **3a** more active than **2a**? The most critical difference between the two compounds is in their stability. The covalent bond between gold and NHC is more stable than the covalent bond constructed between silver and NHC. Silver seems to be worse than gold for NHC ← metal π-back donation [3]. This fact makes **2a** more reactive to free compounds, yielding more interactions with other molecules in the environment than **3a** complexes. Therefore, to compensate these interactions with non-target molecules, a larger number of **2a** complexes are needed, while **3a** complexes seem to go straight to the membranes.

Finally, the results obtained from the MTT assay are found to be in agreement with the low toxicity at concentrations of their MIC and MBEC values between 0.5 to 5.0 µM, and preliminary *ex vivo* toxicity studies demonstrated very low toxicity on the eye when applied externally. Thus, it may prove useful as an external eye drop therapy in patients suffering with keratitis. Similar studies are in progress with some determining the molecular level of interaction with binding modes to bacterial and fungal membrane lipids while others are exploring the synthesis of other gold complexes with different halide groups to enable higher water solubility and lower toxicity. Nevertheless, data reported here are extremely promising, and the reported salts could probably be used in the near future as an important tool to control keratitis-associated eye infection causing multi drug resistant pathogens.

## Supporting Information

Figure S1 Ortep View (40% probability, H removed for clarity) of single crystal X-ray structure of ligand 3,3′-(p-phenylenedimethylene) bis{1-(2- methyl-allyl)imidazolium} bromide(**1a**). Pertinent bond lengths (A°) and angles (°): N1-C7 = 1.320(9), N2-C7 = 1.325(9), bond angles (^o^) N1-C7-N2 = 108.5(6)].
(DOC)

Figure S2 Ortep View (30% probability, H removed for clarity) of single crystal X-ray crystallographic structure of complex (**2a**), Pertinent bond lengths (A°) and angles (°): N5-C29 = 1.33(3), N6-C29 = 1.36(3), C29-Ag3 = 2.08(2), Ag3-Br2 = 2.439(2), N7-C40 = 1.30(3), N8-C40 = 1.34(3), C40-Ag4 = 2.09(2), Ag4-Br4 = 2.409(4), Ag4-Ag1 = 3.084(3), bond angles(^o^): N5-C29-N6 = 104.0(2), C29-Ag3-Br2 = 171.7(7), N7-C40-N8 = 107.0(2), C40-Ag4-Br4 = 172.2(7), C40-Ag4-Ag1 = 97.4(6),Br4-Ag4-Ag = 87.11(12)].
(DOC)

Figure S3 ORTEP View (H atoms have been removed for clarity) of optimized structure of complex (**3a**), Pertinent bond lengths (A°) and angles (°): N55-C58 = 1.31818, N54-C58 = 1.38648, C58-Au111 = 2.06414, Au111-Br107 = 2.43997, N35-C39 = 1.33557, N34-C39 = 1.32855, C39-Au110 = 2.10169, Au110-Br2 = 2.41073, Au110-Au112 = 3.08415, bond angles (^o^): N55-C58-N56 = 102.87193, C58-Au111-Br107 = 171.45808, N34-C39-N35 = 107.52845,C39-Au110-Br2 = 171.45563, C39-Au110-Au112 = 97.47505, Br2-Au110-Au112 = 87.05457]
(DOC)

Figure S4 Ortep View of optimized structure of complex (**2b**), Pertinent bond lengths (A°) and angles (°): N55-C54 = 1.37564, N56-C54 = 1.38058, C54-Ag105 = 2.12572, N6-C1 = 1.38045, N5-C1 = 1.37665, C1-Ag105 = 2.12645, bond angles (^o^): N55-C54-N56 = 104.25472, N5-C1-N6 = 104.27794, C1-Ag105-C55 = 179.81391]
(DOC)

Figure S5 ORTEP View (40% probability, H and PF_6_ removed for clarity) of single crystal X-ray crystallographic structure of complex (**3b**), Pertinent bond lengths (A°) and angles (°): C18-Au1 = 2.016(7), C7-Au1 = 2.1264(5), N3-C18 = 1.353(10), N4-C18 = 1.349(10), N2-C7 = 1.331(9), N1-C7 = 1.357(9), bond angles (^o^): N3-C18-N4 = 103.9(7), N2-C7-N1 = 105.7(6), C7-Au1-C18 = 179.1(3)]
(DOC)

Figure S6 Growth kinetics of bacterial strains with 10^8^ CFU/mL in the presence of different concentrations of compound 3a.
(DOC)

Figure S7 Growth kinetics of fungal strains with 10^7^ CFU/mL in the presence of different concentrations of compound 3a.
(DOC)

Figure S8 Dose-dependent cytotoxic activity of complex **3a** and **2a**. Human breast carcinoma cell (MDA-MB-231) (red and violet lines) and non-carcinoma mouse embryo fibroblast cell (3T3) (blue and black lines) were grown *in vitro* in 96-well plates and treated with different concentrations (0.0 to 100 µM) of complex **3a** (blue and violet color) and **2a** (black and red color). The mean of the percentage of inhibition of cell proliferation compare to control (without complex) along with standard deviation of triplicate results are indicated.
(DOC)

Table S1 Crystal and X-ray diffraction data table of synthesized compounds.
(DOC)

Table S2 Important bond parameters for synthesized compounds.
(DOC)

Text S1 Chemical shift (^1^HNMR and^13^CNMR) and Mass (m/z) data of all synthesized compounds.
(DOC)

Text S2 X-ray crystallographic analysis.
(DOC)

Text S3 Characteristic features of clinical isolates.
(DOC)

Text S4 Procedure for MALDI MS analysis.
(DOC)

## References

[1] Willcox MD (2011) Review of resistance of ocular isolates of *Pseudomonas aeruginosa* and Staphylococci from keratitis to ciprofloxacin, gentamicin and cephalosporins. Clin Exp Optom 94: 161–168. 21083760 10.1111/j.1444-0938.2010.00536.x

[2] Sengupta J, Saha S, Khetan A, Sarkar SK, Mandal SM (2012) Effects of lactoferricin B against keratitis-associated fungal biofilms. J Infect Chemother 18: 698–703. 22410856 10.1007/s10156-012-0398-3

[3] Mellon MG, Benbrook C, Benbrook KL (2001) Hogging It! Estimates of Antimicrobial Abuse in Livestock (Cambridge, MA: Union of Concerned Scientists). Available: http://www.ucsusa.org/food_and_environment/antibiotics_and_food/hogging-it-estimates-of-antimicrobial-abuse-in-livestock.html. Accessed 2008 Mar 12.

[4] Tenover FC (2006) Mechanisms of Antimicrobial Resistance in Bacteria. Am J Med 119: S3–S10. 10.1016/j.amjmed.2006.03.011 16735149

[5] Roymahapatra G, Mandal SM, Porto WF, Samanta T, Giri S, et al (2012) Pyrazine functionalized Ag(I) and Au(I)-NHC complexes are potential antibacterial agents. Curr Med Chem19: 4184–4193. 10.2174/092986712802430090 22680631

[6] Hindi KM, Panzner MJ, Tessier CA, Cannon CL, Youngs WJ (2009) The medicinal applications of imidazolium carbene-metal complexes. Chem Rev 109: 3859–3884. 19580262 10.1021/cr800500u PMC2763352

[7] Hermann WA (2002) N-Heterocyclic carbene as ligands for metal complexes- challenging phsphene ligands in homogenious catalysts. Angew Chem Int Ed 41: 1290–1309.

[8] Knapp AR, Panzner MJ, Medvetz DA, Wright BD, Tessier CA, et al (2010) Synthesis and antimicronial studies of silver N-heterocyclic carbene complexes bearing a methyl benzoate substituent. Inorganica Chim.Acta 364: 125–131. 21218156 10.1016/j.ica.2010.08.008 PMC3014616

[9] Huang W, Zhang,R, Zou G, Tang J, Sun J (2007) An iodide/anion exchange route to benzimidazolylidene silver complexes from benzimidazolium iodide: Crystal structures of N,N′-dibutylbenzimidazolylidene silver chloride, bromide, cyanide and nitrate. J Organomet Chem 692: 3804–3809.

[10] Schuster O, Yang L, Raubenheimer HG, Albrecht M (2009) Beyond conventional N-heterocyclic carbenes: abnormal, remote, and other classes of NHC ligands with reduced heteroatom stabilization. Chem Rev 109: 3445–3478. 19331408 10.1021/cr8005087

[11] Lillo V, Mas-Marzá E, Segarra AM, Carbó JJ, Bo C, et al (2007) Palladium-NHC complexes do catalyse the diboration of alkenes: mechanistic insights Chem Commun. 32: 3380–3382. 10.1039/b705197b 18027432

[12] Hahn EF, Heidrich B, Hepp A, Pape T (2007) Coordination compounds of N,N′-olefin functionalized imidazolin-2-ylidenes. J Organomet Chem 692: 4630–4638.

[13] Sheldrick GM (1997) SHELX76, Programme for refinement of crystal structures,University of Gottingen, Germany. Available: http://shelx.uni-ac.gwdg.de/SHELX/.

[14] Gaussian 03, Revision E.01, (2004) Gaussian, Inc., Wallingford CT.

[15] Saha S, Banerjee D, Khetan A, Sengupta J (2009) Epidemiological profile of fungal keratitis in urban population of West Bengal, India. Oman J Ophthalmol 2: 114–118. 20927207 10.4103/0974-620X.57310 PMC2903915

[16] Clinical and Laboratory Standards Institute Performance standards for antimicrobial susceptibility testing (2007) Seventeenth informational supplement Document M100-S17. CLSI, Wayne, PA: CLSI.

[17] Mitchell BM, Wu TG, Jackson BE, Wilhelmus KR (2007) *Candida albicans* strain-dependent virulence and Rim13p-mediated filamentation in experimental keratomycosis. Invest Ophthalmol Vis Sci 48: 774–780. 17251477 10.1167/iovs.06-0793

[18] Croes S, Deurenberg RH, Boumans ML, Beisser PS, Neef C, et al (2009) *Staphylococcus aureus* biofilm formation at the physiologic glucose concentration depends on the *S. aureus* lineage. BMC Microbiol 9: 229–237. 19863820 10.1186/1471-2180-9-229 PMC2774858

[19] Sahu K, Bansal H, Mukherjee C, Sharma M, Gupta PK (2009) Atomic force microscopic study on morphological alterations induced by photodynamic action of Toluidine Blue O in *Staphylococcus aureus* and *Escherichia coli* . J Photochem Photobiol B 96: 9–16. 19423358 10.1016/j.jphotobiol.2009.03.008

[20] Jo S, Lim JB, Klauda JB, Im W (2009) CHARMM-GUI Membrane Builder for Mixed Bilayers and Its Application to Yeast Membranes. Biophys J 9: 50–58. 10.1016/j.bpj.2009.04.013 PMC2711372 19580743

[21] Murzyn K, Rog T, Pasenkiewicz-Gierula T (2005) Phosphatidylethanolamine-phosphatidylglycerol bilayer as a model of the inner bacterial membrane. Biophys J 88: 1091–1103. 15556990 10.1529/biophysj.104.048835 PMC1305115

[22] Bikadi Z, Hazai I, Malik D, Jemnitz K, Zsuzsa V, et al (2011) Predicting p-glycoprotein-mediated drug transport based on support vector machine and three-dimensional crystal structure of p-glycoprotein. Plos One 6(10): e25815. 21991360 10.1371/journal.pone.0025815 PMC3186768

[23] Lu P, Hontecillas R, Horne WT, Carbo A, Viladomiu M, et al (2012) Computational modeling-based discovery of novel classes of anti-inflammatory drugs that target lanthionine synthetase c-like protein 2. PLoS One 7(4): e34643. 22509338 10.1371/journal.pone.0034643 PMC3324509

[24] Platania CBM, Salomone S, Leggio GM, Drago F, Bucolo C (2012) Homology modeling of dopamine D_2_ and D_3_ receptors: molecular dynamics refinement and docking evaluation. PLoS One 7(9): e44316. 22970199 10.1371/journal.pone.0044316 PMC3435408

[25] Mandal SM, Migliolo L, Das S, Mandal M, Franco OL, et al (2012) Identification and characterization of a bactericidal and proapoptotic peptide from *Cycas revoluta* seeds with DNA binding properties. J Cell Biochem 113(1): 184–193. 21882228 10.1002/jcb.23343

[26] Barnes NA, Brisdon AK, Brown WFR, Cross WI, Crossley IR, et al (2011) Synthesis of gold(I) fluoroalkyl and fluoroalkenyl-substituted phosphine complexes and factors affecting their cryst7al packing. Dalton Trans 40: 1743–1750. 21132182 10.1039/c0dt01014f

[27] Poole K (2004) Efflux-mediated multiresistance in Gram-negative bacteria. Clin Microbiol Infect 10: 12–26. 10.1111/j.1469-0691.2004.00763.x 14706082

[28] Parthasarathi R, Padmanabhan J, Subramanian V, Maiti B, Chattaraj PK (2003) Chemical reactivity profiles of two selected polychlorinated biphenyls. J Phys Chem A 107: 10346–10352.

[29] Arulmozhiraja S, Selvin PC, Fujiti TJ (2002) Structures, potential energy curves and torsional barrier heights for selected polychlorinated biphenyls: a density functional theory study. J Phys Chem A 106: 1765–1769.

[30] Berners-Price SJ, Mirabelli CK, Jhonson RK, McCabe LF, Sung CM, et al (1986) *In Vivo* antitumor activity and *in vitro* cytotoxic properties of bis[1,2-bis(diphenylphosphino)ethane]gold(i) chloride. Cancer Res 46: 5486–5493. 3756897

[31] Özdemir I, Denizci A, Öztürk HT, Çetinkaya B (2004) Synthetic and antimicrobial studies on new gold(I) complexes of imidazolidin-2-ylidenes. Appl Organomet Chem 18: 318–322.

