# Supplementary material for: N, N′-Olefin Functionalized Bis-Imidazolium Gold(I) Salt Is an Efficient Candidate to Control Keratitis-Associated Eye Infection
Source: PLoS One. 2013 Mar 15;8(3):e58346. doi: 10.1371/journal.pone.0058346 (PMC3598898; doi:10.1371/journal.pone.0058346)
Supplement: Figure S2 — Ortep View (30% probability, H removed for clarity) of single crystal X-ray crystallographic structure of complex (2a), Pertinent bond lengths (A°) and angles (°): N5-C29 = 1.33(3), N6-C29 = 1.36(3), C29-Ag3 = 2.08(2), Ag3-Br2 = 2.439(2), N7-C40 = 1.30(3), N8-C40 = 1.34(3), C40-Ag4 = 2.09(2), Ag4-Br4 = 2.409(4), Ag4-Ag1 = 3.084(3), bond angles(o): N5-C29-N6 = 104.0(2), C29-Ag3-Br2 = 171.7(7), N7-C40-N8 = 107.0(2), C40-Ag4-Br4 = 172.2(7), C40-Ag4-Ag1 = 97.4(6),Br4-Ag4-Ag = 87.11(12)]. (DOC) [file pone.0058346.s002.doc]

**Figure S2.**
