# Supplementary material for: N, N′-Olefin Functionalized Bis-Imidazolium Gold(I) Salt Is an Efficient Candidate to Control Keratitis-Associated Eye Infection
Source: PLoS One. 2013 Mar 15;8(3):e58346. doi: 10.1371/journal.pone.0058346 (PMC3598898; doi:10.1371/journal.pone.0058346)
Supplement: Figure S3 — ORTEP View (H atoms have been removed for clarity) of optimized structure of complex (3a), Pertinent bond lengths (A°) and angles (°): N55-C58 = 1.31818, N54-C58 = 1.38648, C58-Au111 = 2.06414, Au111-Br107 = 2.43997, N35-C39 = 1.33557, N34-C39 = 1.32855, C39-Au110 = 2.10169, Au110-Br2 = 2.41073, Au110-Au112 = 3.08415, bond angles (o): N55-C58-N56 = 102.87193, C58-Au111-Br107 = 171.45808, N34-C39-N35 = 107.52845,C39-Au110-Br2 = 171.45563, C39-Au110-Au112 = 97.47505, Br2-Au110-Au112 = 87.05457] (DOC) [file pone.0058346.s003.doc]

**Figure S3**.
