# Supplementary material for: N, N′-Olefin Functionalized Bis-Imidazolium Gold(I) Salt Is an Efficient Candidate to Control Keratitis-Associated Eye Infection
Source: PLoS One. 2013 Mar 15;8(3):e58346. doi: 10.1371/journal.pone.0058346 (PMC3598898; doi:10.1371/journal.pone.0058346)
Supplement: Figure S4 — Ortep View of optimized structure of complex (2b), Pertinent bond lengths (A°) and angles (°): N55-C54 = 1.37564, N56-C54 = 1.38058, C54-Ag105 = 2.12572, N6-C1 = 1.38045, N5-C1 = 1.37665, C1-Ag105 = 2.12645, bond angles (o): N55-C54-N56 = 104.25472, N5-C1-N6 = 104.27794, C1-Ag105-C55 = 179.81391] (DOC) [file pone.0058346.s004.doc]

**Figure S4.**
