# Supplementary material for: N, N′-Olefin Functionalized Bis-Imidazolium Gold(I) Salt Is an Efficient Candidate to Control Keratitis-Associated Eye Infection
Source: PLoS One. 2013 Mar 15;8(3):e58346. doi: 10.1371/journal.pone.0058346 (PMC3598898; doi:10.1371/journal.pone.0058346)
Supplement: Figure S6 — Growth kinetics of bacterial strains with 108 CFU/mL in the presence of different concentrations of compound 3a. (DOC) [file pone.0058346.s006.doc]

**Figure S6.**
