# Supplementary figures and images for: N, N′-Olefin Functionalized Bis-Imidazolium Gold(I) Salt Is an Efficient Candidate to Control Keratitis-Associated Eye Infection
Source: PLoS One. 2013 Mar 15;8(3):e58346. doi: 10.1371/journal.pone.0058346 (PMC3598898; doi:10.1371/journal.pone.0058346)

**Figure S8**.


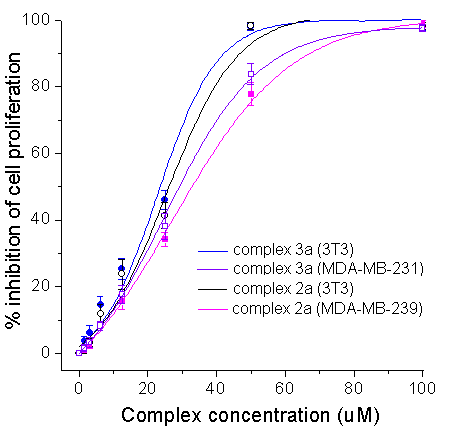

Supplement: Figure S8 — Dose-dependent cytotoxic activity of complex 3a and 2a. Human breast carcinoma cell (MDA-MB-231) (red and violet lines) and non-carcinoma mouse embryo fibroblast cell (3T3) (blue and black lines) were grown in vitro in 96-well plates and treated with different concentrations (0.0 to 100 µM) of complex 3a (blue and violet color) and 2a (black and red color). The mean of the percentage of inhibition of cell proliferation compare to control (without complex) along with standard deviation of triplicate results are indicated. (DOC) [file pone.0058346.s008.doc]
