# Supplementary material for: N, N′-Olefin Functionalized Bis-Imidazolium Gold(I) Salt Is an Efficient Candidate to Control Keratitis-Associated Eye Infection
Source: PLoS One. 2013 Mar 15;8(3):e58346. doi: 10.1371/journal.pone.0058346 (PMC3598898; doi:10.1371/journal.pone.0058346)
Supplement: Table S1 — Crystal and X-ray diffraction data table of synthesized compounds. (DOC) [file pone.0058346.s009.doc]

**Table S1**.

| Empirical formula | C_22_H_28_Br_2_N_4_ | C_22_H_26_N_4_Br_2_Ag_2_ | C_44_ H_52_ Au_2_ F_12_ N_8_ P_2_ |
| --- | --- | --- | --- |
| Formula Weight | 508.30 | 722.03 | 1376.81 |
| Wavelength (Å) | 0.71073 | 0.71073 | 0.71073 |
| Crystal system | Monoclinic | Triclinic | Triclinic |
| Space group | P21/n | P-1 | P-1 |
| Temperature | 296(2) | 296(2) | 150(2) |
| Unit celldimensions |  | | |
| a (Å) | 7.2570(2) | 15.197(10) | 11.295(3) |
| b (Å) | 10.6742(3) | 10.404(7) | 11.582(2) |
| c (Å) | 15.9962(5) | 16.387(11) | 12.331(3) |
| α (°) | 90.00 | 89.953(8) | 107.616(2) |
| β (°) | 100.1040(10) | 108.739(7) | 97.707(4) |
| γ (°) | 90.00 | 89.995(7) | 118.777(3) |
| Volume (Å3 ) | 1219.89(6) | 2454(3) | 1269.8(5) |
| Z | 2 | 2 | 1 |
| Density (calculated) Mg/m3 | 1.384 | 1.955 | 1.801 |
| Crystal size mm3 | 0.17×0.11×0.06 | 0.32×0.25×0.12 | 0.23×0.16×0.09 |
| Absorption coeffi. (µ) | 3.336 | 4.869 | 5.917 |
| F(000) | 516 | 1400 | 668 |
| θ range for data collection (°) | 1.5 – 25.0 | 1.5 – 25.0 | 1.5 – 25.0 |
| Index ranges | -8<h<8; -12<k<12; -18<l<18 | -15<h<15;-10<k<10; -17<l<17 | -13<h<13;-13<k<13; -14<l<14 |
| Reflections collected | 2153 | 2981 | 4463 |
| Independent reflections | 2044 | 2043 | 3731 |
| Goodness-of -fit on F2 | 1.196 | 1.083 | 1.024 |
| R1(observed / all data) | 0.0445 | 0.1551 | 0.0508 |
| wR2 (observed / all data) | 0.1709 | 0.2272 | 0.1025 |
