# Supplementary material for: N, N′-Olefin Functionalized Bis-Imidazolium Gold(I) Salt Is an Efficient Candidate to Control Keratitis-Associated Eye Infection
Source: PLoS One. 2013 Mar 15;8(3):e58346. doi: 10.1371/journal.pone.0058346 (PMC3598898; doi:10.1371/journal.pone.0058346)
Supplement: Table S2 — Important bond parameters for synthesized compounds. (DOC) [file pone.0058346.s010.doc]

**Table S2.**

| **Parameters** | **Ligand-1a (XRD data)** | **Complex-2a (XRD data)** | **Complex-3a (Theoretical)** | **Complex-2b (Theoretical)** | **Complex-3b (XRD data)** |
| --- | --- | --- | --- | --- | --- |
| Relative bond distances (A^0^) | N1-C7 = 1.320(9)  N2-C7 = 1.325(9) | N5-C29 =1.33(3)  N6-C29 =1.36(3)  C29-Ag3 =2.08(2) .  Ag3-Br2 =2.439(2)  N7-C40 =1.30(3) .  N8-C40 =1.34(3) .  C40-Ag4 = 2.09(2)  Ag4-Br4 = 2.409(4)  Ag4-Ag1 = 3.084(3) | N55-C58 =1.31818  N54-C58 =1.38648  C58-Au111 = 2.06414  Au111-Br107 =2.43997  N35-C39 =1.33557  N34-C39 =1.32855  C39-Au110 = 2.10169  Au110-Br2 = 2.41073  Au110-Au112 = 3.08415 | N55-C54 =1.37564  N56-C54 =1.38058  C54-Ag105 = 2.12572  N6-C1 = 1.38045  N5-C1 = 1.37665  C1-Ag105 = 2.12645 | N3-C18 =1.353(10)  N4-C18 =1.349(10)  C18-Au1 = 2.016(7)  N2-C7 = 1.331(9)  N1-C7 = 1.357(9)  C7-Au1 = 2.12645 |
| Relative bond angles (^0^) | N1-C7-N2 = 108.5(6) | N5-C29-N6 = 104.0(2)  C29-Ag3-Br2 =171.7(7)  N7-C40-N8 = 107.0(2)  C40-Ag4-Br4 = 172.2(7)  C40-Ag4-Ag1 = 97.4(6)  Br4-Ag4-Ag1 = 87.11(12) | N55-C58-N56 = 102.87193  C58-Au111-Br107 =171.45808  N34-C39-N35 = 107.52845  C39-Au110-Br2 = 171.45563  C39-Au110-Au112 = 97.47505  Br2-Au110-Au112 = 87.05457 | N55-C54-N56 = 104.25472  N5-C1-N6 = 104.27794  C1-Ag105-C55 =179.81391 | N3-C18-N4 = 103.9(7)  N2-C7-N1 = 105.7(6)  C7-Au1-C18 =179.1(3) |
