# Supplementary material for: N, N′-Olefin Functionalized Bis-Imidazolium Gold(I) Salt Is an Efficient Candidate to Control Keratitis-Associated Eye Infection
Source: PLoS One. 2013 Mar 15;8(3):e58346. doi: 10.1371/journal.pone.0058346 (PMC3598898; doi:10.1371/journal.pone.0058346)
Supplement: Text S1 — Chemical shift (1HNMR and13CNMR) and Mass (m/z) data of all synthesized compounds. (DOC) [file pone.0058346.s011.doc]

**Text S1:**

**Synthesis of 3,3'-(p-phenylenedimethylene) bis{1-(2- methyl-allyl)imidazolium} bromide(1a):** ^1^HNMR(300MHz, DMSO- d_6_ ): δ = 9.47 (s,1H,a), 7.90 (s,1H,b),7.77 (s,1H,c),7.51 ( 2H,h), 5.51( s,2H,g), 5.05(s,1H,e), 4.84(s,1H,e), 4.84(s,2H,d), 1.68, (_S_,3H,f). ^13^CNMR(75MHz, DMSO-d_6_): δ = 139.3(a), 136.6(b),135.4(c),128.9 (h), 123.2(i), 122.7(g), 114.9(d) , 54.1(j), 51.4(e), 19.5(f) . MALDI Mass: (m/z) values are 348.18, 347.18. Anal.Calc. for C_22_H_28_N_4_Br_2_ (M_w_  = 508): C,51.96; H ,5.51;N,11.02% Found: C,51.94;H,5.50;N,10.97%.

**Synthesis of 3,3'-(p-phenylenedimethylene) bis{1-(2- methyl-allyl)imidazolium} hexafluorophosphate salt (1b):** ^1^HNMR(300MHz, DMSO-d_6_ ): δ = 9.25 (s,1H,a), 7.86 (s,1H,b),7.74 (s,1H,c), 7.47,(2H,h), 5.51(s,2H,g), 7.05(s,1H,e), 4.84{s,1H,e),4.84Is,2H,d), 1.68,(s,3H,f). ^13^CNMR(75MHz, DMSO-d_6_): δ = 137.5(a), 135.7(b), 134.8(c), 128.5(h), 122.9(i), 122.6(g), 114.2(d) , 53.8(j), 51.0(e), 19.3(f) . MALDI Mass: (m/z) values are 491.91 and 347.18. Anal.Calc. for C_22_H_28_N_4_(PF_6_)_2_ (M_w_ =638): C, 41.37; H, 4.39;N,8.77%; Found : C,41.34;H,4.37;N,8.76%.

**Synthesis of 3,3'-(p-phenylenedimethylene) bis{1-(2- methyl-allyl)imidazoline}silver bromide (2a):** ^1^HNMR (300MHz, DMSO-d_6_): δ = 7.54 (s,1H,b), 7.42 (s,1H,c), 7.14 (s,2H,h), 5.30 (s,2H,g), 4.90(s,1H,e), 4.70(s,1H,e) ,4.70(s,2H,d), 1.68,(s,3H,f). ^13^CNMR(75MHz, DMSO- d_6_): δ = 178.2(a), 135.9(b),135.3(c),128.6 (h), 123.1(i), 122.4(g), 115.3(d) , 54.9(j), 52.3(e), 20.2(f). MALDI Mass: (m/z) values are 641.38, 561.58, and 454.10. Anal.Calc. for C_22_H_26_Ag_2_N_4_Br_2_: (M_w_ =722): C,36.56; H,3.301; N,7.76%. Found: C,36.54;H,3.29; N,7.74 %.

**Synthesis of 3,3’-(p-phenylenedimethylene) bis{1-(2- methyl-allyl)imidazoline}gold(I) bromide (3a):** ^1^HNMR (300MHz, DMSO-d_6_): δ = 7.56 {d,1H,b(J=2Hz)}, 7.43{d,1H,c,(J=2.4Hz)}, 7.34(s,2H,h), 5.36 (s,2Hg), 4.95(s,1H,e), 4.70(s,2H,d), 4.68(s,1H, e), 1.68,(s,3H,f). ^13^CNMR(75MHz, DMSO- d_6_): δ = 179.4(a), 135.4(b),134.9(c),128.4 (h), 122.8(i), 122.5(g), 115.1(d) , 54.4(j), 52.2(e), 19.9(f). MALDI Mass: (m/z) values are 837.68, 757.78 and 560.88. (M_w_  = 900): C,29.33; H,2.88;N=6.22%; Found: C,28.97; H,2.87; N,6.19 %.

**Synthesis of 3,3’-(p-phenylenedimethylene) bis{1-(2- methyl-allyl)imidazoline}silver hexafluorophosphate (2b):** ^1^HNMR (300MHz, DMSO-d_6_): δ = 7.57 {d,1H,b(J=5.2Mz)}, 7.39 {d,1H,c(J=5.2Mz), 6.99(s,2H,h), 5.29 (s,2H,g), 4.88(s,1H,e) , 4.74(s,2H,d), 4.74(s,1H,e), 1.66,(s,3H,f). ^13^C NMR(75MHz, DMSO- d_6_): δ = 175.3(a), 135.2(b),134.7(c),128.3 (h), 122.9(i), 122.2(g), 115.1(d) , 54.2(j), 52.0(e), 19.9(f). MALDI Mass: (m/z) values are-952.48 and 807.78. Anal.Calc. for C_44_H_52_Ag_2_N_8_(PF_6_)_2_ : (M_w_ ,1196) : C,44.15; H,4.34; N,9.36%. Found C, 44.11; H, 4.33; N, 9.34%.

**Synthesis of 3,3'-(p-phenylenedimethylene) bis{1-(2- methyl-allyl)imidazoline}gold(I) hexafluorophosphate (3b):** ^1^HNMR (300MHz, DMSO-d_6_): δ = 7.62 {d,1H,b (J=2Hz)}, 7.49 {d,1H,c(J=2.4Hz), 7.12 {d,2H,h(J=12Hz)}, 5.36 (s,2H,g), 4.93{s,1H,e), 4.78(s,2H,d), 4.73(s,1H,e), 1.66,(s,3H,f). ^13^CNMR(75MHz, DMSO-d_6_): δ = 177.5(a), 135.1(b),134.7(c),128.2 (h), 122.5(i), 122.3(g), 115.0(d) , 54.5(j), 52.0(e), 19.8(f). MALDI Mass: (m/z) values are 1230.75 and 1086.03. Anal.Calc. for C_44_H_52_Au_2_N_8_(PF_6_)_2_ : (M_w_ ,1376 ): C,38.37; H=3.78, N=`8.14. Found C=38.35, H=3.77, N=8.14%.
