# Supplementary material for: N, N′-Olefin Functionalized Bis-Imidazolium Gold(I) Salt Is an Efficient Candidate to Control Keratitis-Associated Eye Infection
Source: PLoS One. 2013 Mar 15;8(3):e58346. doi: 10.1371/journal.pone.0058346 (PMC3598898; doi:10.1371/journal.pone.0058346)
Supplement: Text S2 — X-ray crystallographic analysis. (DOC) [file pone.0058346.s012.doc]

**Text S2:**

In order to understand the ligand coordination geometry, single crystals were developed using the suitable crystal for X-ray data collection. Vapor diffusion of diethyl ether into methanolic solution of **1a** developed the colorless crystals. The bond parameters of synthesized compounds are listed in **Table S2**. The molecule possessed N(1)-C(7), N(2)-C(7), distances1.320(9) and 1.325(9) A° respectively, and the procabenic angle N1-C7-N2 = 108.5(6)° was comparable with other imidazolium systems. From the solid state structure it was observed that two procarbenic carbons remain on the opposite side of the bridging benzyl group. Vapor diffusion of Et_2_O into an dichloromethane solution of **2a** yielded a sample of a lower crystalloid dinuclear [Ag_2_(**1a**-2H)Br)_2_] complex and bonding motif of bis-carbene complex with olifinic system. The Ag-C_carbene_ bond distances C(40)-Ag(4) = 2.09(2), C(29)-Ag(3) =2.08(2) A° were consistent with the reported (NHC-Ag-X, X = Cl, Br etc) monocarbene system and within the sum of van der Waals radii of Ag(I) and carbon. The Ag-Br bond distances were Ag3-Br2 = 2.439(2), Ag4-Br4 = 2.409(4) Å. The molecule showed Ag---Ag bond length 3.084(3) Å within the reported distances, The C—Ag—Br bond angle varies from 170.5(6)– 171.1(7)º *i.e.* almost linear, whereas the Ag—Ag—Br bond angle was nearly orthogonal [Br2 Ag3 Ag2 84.99(10)], which indicates that the Ag—Ag bond was perpendicular to C—Ag—Br *i.e.* forming a ‘T’ shape. **2a** formed an infinite chain through Ag–Ag interactions. The structure was optimized through B3LYP. The bond parameters were consistent with **2a**. The Au-C_arbene_ distances varied from 2.06414-2.10169 Å; Au-Br bond distances varied from 2.41073-2.43997Å. The carbenic carbon angles 102.87193-107.52845°were consistent with the reported systems. The Au(I)--Au(I) bond distance 3.08415 Å was comparable with Ag--Ag separation of the same ligand. The structure of **2b** was also optimized. Geometry and bond parameters of the optimized structure were compared with **2a** and **3b**. In the optimized structure of **2b** the bonding linkage (biscarbene) was quite different from that of **2a,** but the Ag-C _carbene_  bond distances [ 2.12572-2.12645 ] and N- C _carbene_  -N bond angles [104.254- 104.277] were comparable with **2a**. The crystals suitable for X-ray diffraction were grown from slow diffusion of a saturated acetonitrile solution of complex **3b** into diethyl ether. The molecule crystallized as triclinic symmetry with P-1 space group and possessed a center of inversion. In the binuclear unit of **3b**, the linear bonding connectivity of Au(I)-C_carbene_ bore the bond lengths Ag(1)-C(7) = 2.1264(5), and Au(1)-C(18) = 2.016(7), Å; comparable with the Ag- C_carbene_ separation in **3a**. The biscarbene C_carbene_– Au - C_carbene_ coordination was almost linear [C(7)-Au(1)-C(18) =179.1(3)°] .The Au-C bond distances varied between 2.016(7) and 2.1264(5) Å, which was comparable with other known biscarbene Au(I)-NHC linkages and within the sum of van der Wall radii of gold and carbon atoms^21^. The carbenic angle was reduced after complexation [N2-C7-N1 = 105.7(6) and N3-C18-N4 = 103.9(7)°], becoming smaller than the corresponding proligand [*N1-C7-N2 = 108.5(6)*]. There was no Au(I)--- Au(I) interaction (8.824 Å) in the metallocycle. The ORTEP drawing of all synthesized compounds is shown in Figure S1-S5.
