# Supplementary material for: N, N′-Olefin Functionalized Bis-Imidazolium Gold(I) Salt Is an Efficient Candidate to Control Keratitis-Associated Eye Infection
Source: PLoS One. 2013 Mar 15;8(3):e58346. doi: 10.1371/journal.pone.0058346 (PMC3598898; doi:10.1371/journal.pone.0058346)
Supplement: Text S3 — Characteristic features of clinical isolates. (DOC) [file pone.0058346.s013.doc]

**Text S3:**

***Aspergillus fumigatus***

**Macroscopic morphology:**

On PDA colonies show typical blue green surface pigmentation with a suede like surface consisting of adense felt of conidiophores.

**Microscopic morphology:**

Conidial heads are typical columnar (upto 400×500 µm but often much shorter and smaller) and uniseriate. Conidiophores are short, smooth walled and have conical shaped terminal vesicles which support a single row of phialides on the upper two thirds of the vesicle. Conidia are produced in basipetal succession forming long chains and are globose to subglobose, green and round walled. Electron microscopic (SEM) images of the spores showed the above feature which confirms the species as *Aspergillus fumigates*.


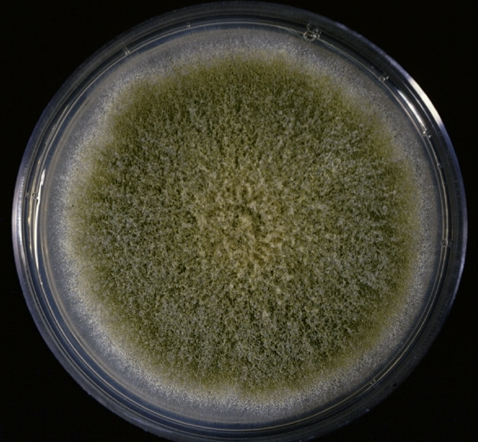

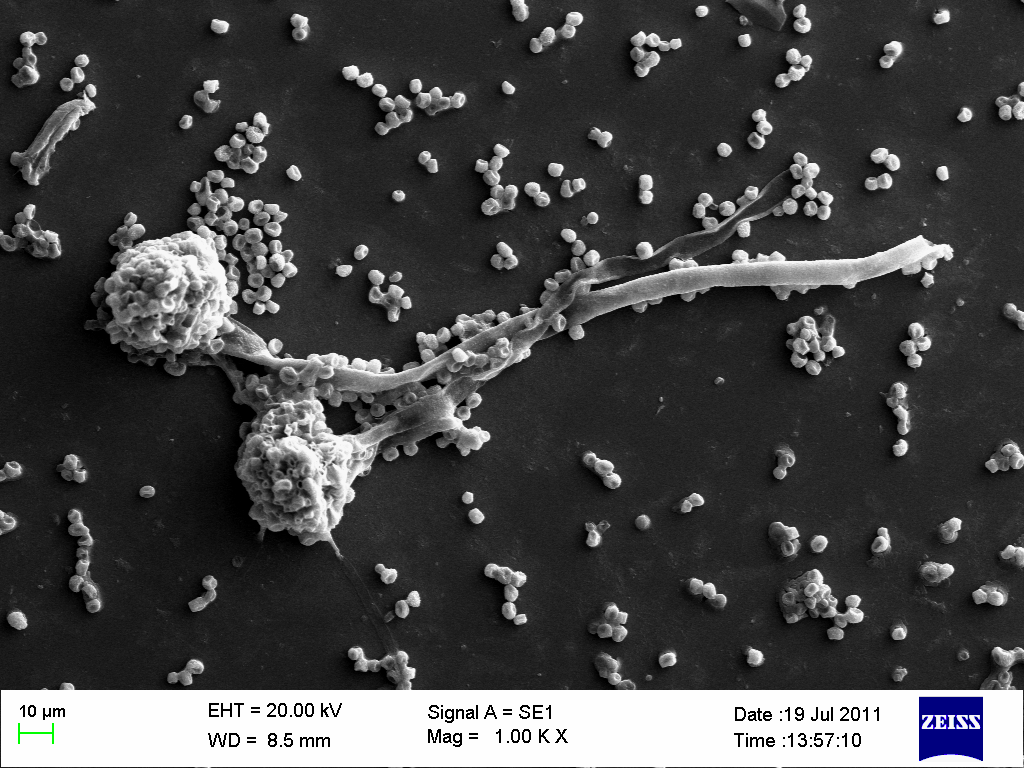


Figure: Isolate was grown on PDA agar medium (left) and SEM images spore with sporangiophore.

***Candida albicans***

**Macroscopic Morphology**

*Candida albicans* grows well on PDA, SDA and other bacteriological media. Convex entire margin non mucoid smooth texture of cream colored pasty colonies usually appear after 24-48 hrs incubation at 35-37 C. the colonies have a distinctive yeast smell.

**Microscopic Morphology**

The round oval shaped budding cells can be easily seen by direct microscopy in stained or unstained preparation. It produces true germ tube when incubated in serum for 2-3 hrs at 37C


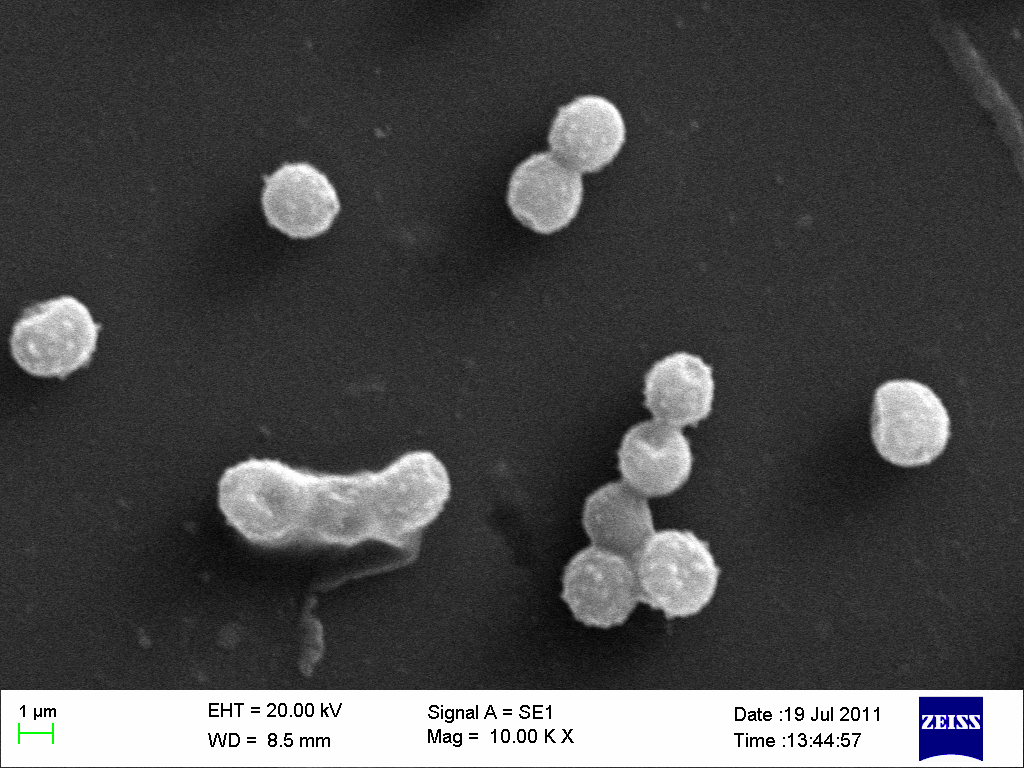

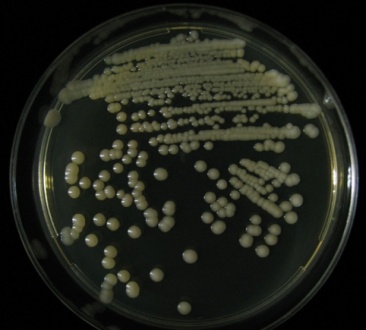


Figure: Isolate was grown on PDA agar medium (left) and SEM images of individual cell.

**Antimicrobial sensitivity test of clinically isolated bacteria***

|  | *S. aureus* | *P. aeruginosa* |
| --- | --- | --- |
| Antibiotics | Zone of Inhibition (mm) | Zone of Inhibition (mm) |
| **Ciprofloxacin (CF)** | 23 | 32 |
| **Gatifloxacin (GF)** | 28 | 30 |
| **Gentamicin (G)** | 30 | 28 |
| **Lomefloxacin (LO)** | 10 | 22 |
| **Tobramycin (TB)** | 32 | 25 |
| **Amikacin (AK)** | 20 | 21 |
| **Ofloxacin (OF)** | 20 | 22 |
| **Azithromycin (AZ)** | 25 | 20 |
| **Cephaloridine (CR)** | 00 | 00 |
| **Chloramphenicol (C)** | 30 | 20 |
| **Rifammycin (RF)** | 15 | 18 |
| **Cefazolin (CZ)** | 20 | 15 |
| **Moxifloxacin (MO)** | 28 | 32 |
| **Imepenem (I)** | 00 | 20 |
| **Tricarcillin (TI)** | 00 | 15 |
| **Cefalexin (CP)** | 00 | 00 |
| **Amoxycillin (AM)** | 10 | 20 |
| **Cefadroxyl (CQ)** | 28 | 00 |
| **Meropenam (ME)** | 20 | 10 |

*****Clinically correlated antibiotic disc were purchased from Himedia Co., India. Red color data are indicating the resistant to antibiotics.
