# Supplementary material for: N, N′-Olefin Functionalized Bis-Imidazolium Gold(I) Salt Is an Efficient Candidate to Control Keratitis-Associated Eye Infection
Source: PLoS One. 2013 Mar 15;8(3):e58346. doi: 10.1371/journal.pone.0058346 (PMC3598898; doi:10.1371/journal.pone.0058346)
Supplement: Text S4 — Procedure for MALDI MS analysis. (DOC) [file pone.0058346.s014.doc]

**Text S4:**

The complexes were re-suspended in acetonitrile and four microliters of individual complex solution was mixed with 4 µL of matrix (dihydroxy benzoic acid, 10 mg.mL^-1^) and 1.0 µL of this mixture solution was spotted onto the MALDI 100 well stainless steel sample plate and allowed to air dry prior to the MALDI analysis. To obtain MALDI mass spectra, a Voyager time-of-flight mass spectrometer (Applied Biosystem, USA), equipped with 337 nm N_2_ laser was used and operated in accelerating voltage 20 kV. The spectra were recorded in the positive ion linear mode. Reproducibility of the spectrum was checked 5 times from separately spotted samples.
